# Impact of Two-Stage Weaning and Bovine-Appeasing Substance on Growth, Temperament, Pasture Behavior, and Immune System of Nellore Calves

**DOI:** 10.3390/ani15111640

**Published:** 2025-06-03

**Authors:** Mariana Santos, Dalton Mendes de Oliveira, Matheus Rodrigues de Souza, Gabrielly Benevides de Almeida, Aylpy Renan Dutra Santos, Juliano Cesar Castro Belmonte, Fabiana de Andrade Melo-Sterza, André Luiz Julien Ferraz, Marcelo Vedovatto

**Affiliations:** 1 Campus de Aquidauana, Mato Grosso do Sul State University, Aquidauana 79200-000, MS, Brazil; mariana.santos.mszt@gmail.com (M.S.); dmo@uems.br (D.M.d.O.); matheus.rodrigues.zootecnia@gmail.com (M.R.d.S.); gabriellyzootec21@gmail.com (G.B.d.A.); renanufma@hotmail.com (A.R.D.S.); julianobelmonte12@gmail.com (J.C.C.B.); fabiana.sterza@gmail.com (F.d.A.M.-S.); splinter@uems.br (A.L.J.F.); 2 Dean Lee Research and Extension Center, Louisiana State University, Alexandria, LA 71302, USA

**Keywords:** cattle, grazing, nose flap, pheromone, secure cattle

## Abstract

**Simple Summary:**

Weaning is a stressful phase for calves that can lead to weight loss, reduced feed efficiency, behavioral issues, and weakened immunity. Among the promising strategies to reduce weaning stress, the nose flap (NF) stands out, as it gradually decreases suckling behavior. Another alternative is the bovine-appeasing substance (BAS), which induces calmness when detected by the calves’ vomeronasal system. Therefore, this experiment investigated how the use of NFs 14 days before weaning and the administration of BASs at weaning (day 0) affect calves’ growth, temperament, immune function, and pasture behavior during the weaning period. The use of the NF reduced calf weight gain and had negative effects on their behavior in the handling chute. In contrast, BAS administration resulted in increased weight gain and a reduced expression of stress-related behaviors. Therefore, BAS administration reduced the stress by weaning but the use of NFs did not.

**Abstract:**

This experiment evaluated the effects of a nose flap (NF) device and bovine-appeasing substance (BAS) administration on the growth, temperament, immune response, and pasture behavior of calves during weaning. A total of 24 Nellore calves were used in a 2 × 2 factorial arrangement. The factors were the use of an NF or not (NoNF) for 14 d before weaning and the administration of BASs or not (NoBAS) at weaning (d 0). NF vs. NoNF reduced (*p* ≤ 0.01) the average daily gain (ADG; d −7 to 84; −0.289 vs. 0.378 ± 0.13 kg/d) and the time ruminating but increased (*p* ≤ 0.05) the exit score from the chute (d 14 and 28) and the time grazing. BAS vs. NoBAS administration increased (*p* ≤ 0.02) the ADG (d 14 to 84; 0.487 vs. −0.08 ± 0.10 kg/d) and the time grazing, reduced (*p* < 0.01) the time ruminating, and tended to decrease (*p* ≤ 0.10) the exit score (d 7, 14 and 28) and the time vocalizing. Treatments did not affect (*p* ≥ 0.35) serum rabies titer concentration. Thus, the use of NFs reduced growth and increased stress after weaning, while BAS administration increased growth and decreased stress after weaning, altering behavior but not the immune system. According to the results of this experiment, the use of BASs but not NFs is recommended to alleviate weaning stress in beef calves.

## 1. Introduction

Weaning is a critical and stressful transition in the life of beef calves that is often associated with reduced feed intake, weight loss, behavioral disturbances, and compromised immune function [1]. Therefore, identifying effective strategies to mitigate weaning-associated stress is essential for improving animal welfare and performance. Among the proposed alternatives, the use of nose flaps (NFs; i.e., a two-stage weaning method involving anti-suckling devices) has gained attention. These devices physically prevent nursing, promoting a gradual separation from the dam and reducing the abruptness of weaning stress [2].

Another promising approach involves the administration of bovine-appeasing substances (BASs), which are synthetic analogues of natural pheromones. When detected by the calf’s vomeronasal system, BASs can induce a calming effect, modulating behavioral and physiological responses to stress [3,4,5,6]. Recent studies have demonstrated that BASs can attenuate stress responses during weaning, improve behavior and immune parameters, and even promote greater weight gain [3,4,5,6,7].

However, the potential additive or synergistic effects of combining NFs and BASs during the weaning process remain unexplored. To our knowledge, no studies have evaluated the combined use of these strategies in beef calves. Thus, the objective of this study was to investigate the effects of NFs and BASs on post-weaning growth, temperament, grazing behavior, and immune response in Nellore calves. We hypothesized that this combined strategy would improve calf adaptation to weaning by enhancing welfare, immune status, and growth outcomes.

## 2. Materials and Methods

### 2.1. Location and Procedures

The experiment was conducted at the Beef Cattle Farm of the Mato Grosso do Sul State University, Aquidauana University Unit (UEMS/UUA), located in Aquidauana, Mato Grosso do Sul, Brazil. According to the Köppen classification, the climate is tropical savanna with dry winter and summer temperatures that can exceed 35 °C.

### 2.2. Animals, Treatments and Sample Collection

A total of 24 nursing Nellore cows and their 24 calves were used in this experiment. The animals were kept in a grazing system (*Urochloa brizantha* cv. Marandu) with the availability of mineral/vitamin supplement [ProBeef 800; Nutron Animal Nutrition; target intake of 25 g to 35 g/100 kg of body weight (BW)] and water *ad libitum*. Fourteen days (d −14) before weaning (d 0), calves were stratified by BW and sex and randomly assigned to a 2 × 2 factorial arrangement. The first factor was the use of an NF device (Walmur, Porto Alegre, Rio Grande do Sul, Brazil) or not (NoNF) from d −14 to d 0 (weaning), and all animals were kept in a single pasture during this period. The second factor was the BAS administration (Secure Cattle, IRSEA Group, Quartier Salignan, France) or not (NoBAS; saline solution; 0.9% NaCl) on d 0. From this period until d 14, calves were kept separated by BAS treatments in two pastures and then regrouped to a single pasture from d 14 to 84. This management of keeping the animals separated into two groups from d 0 to d 14 was adopted to avoid a possible crossover effect of BASs on the NoBAS calves, since the estimated period of action of BASs is up to 14 d after administration, according to the manufacturer. Both BAS and saline solutions were administered topically in the nuchal area in the amount of 5 mL/animal, as recommended by the manufacturer.

On d −14, a vaccine containing a Pasteur-inactivated virus adsorbed on aluminum hydroxide gel and produced using cell cultures (Raivacel Multi, MSD Saude Animal, Montes Claros, MG, Brazil) was subcutaneously applied to all calves at a dose of 2 mL/animal. Calves were fully weighed on d −14, −7, 0, 7, 14, 28, 56, and 84 to determine the average daily gain (ADG). Animals were not fasted before weighing to avoid the influence of shrink stress-related variables (i.e., temperament, behavior, immune system, etc.).

The temperament of calves was assessed in the chute by three previously trained evaluators (blinded for treatments) on d −14, −7, 0, 7, 14, 28, 56, and 84. The chute score was evaluated using an adaptation of the procedure of Cooke et al. [8], where 1 = calm with no movement; 2 = restless movements; 3 = frequent movement; 4 = constant movement, vocalization, shaking of the chute; and 5 = violent and continuous struggling. The exit scores from the squeeze chute were evaluated according to Baszczak et al. [9] with scores 1 = animals that walked out of the chute, 2 = those that trotted from the chute, and 3 = those that ran or galloped out of the chute.

Blood samples were collected on d −14, −7, 0, 7, 14, 28, 56, and 84 from a jugular vein into one blood collection tube (10 mL; Vacutainer, Becton Dickinson, Franklin Lakes, NJ, USA) without the presence of sodium heparin to obtain serum. After identification of the tubes, the samples were placed in a thermal box with ice and then centrifuged for 15 min at a speed of 1200× *g* for 30 min to separate the serum. On the same day of collection, the serum was stored at −20 °C for later analysis of titer concentration against rabies.

Daily pasture behavior was assessed by visual observation on d −14, −13, −12, −11, −10, −9, −8, −7, −6, −5 −4, −3, 1, 2, 3, 4, 5, 7 and 8 from 0700 h to 1700 h (at 10-min intervals), totaling 10 h of observation per day. The variables evaluated were adapted from Enríquez et al. [10] and are described in Table 1.

The herbage mass was evaluated on d −14, 0, 7, 14, 28, 56 and 84 using the comparative yield method [11], and the samples collected were dried at 55 °C for 5 d and weighed. Forage allowance was calculated as the average herbage mass divided by the average total BW of calves in each pasture. Hand-picked forage samples were also collected on d −14, 0, 7, 14, 28, 56 and 84. Afterward, samples were dried at 55 °C for 5 d and grounded at 1 mm for later chemical composition analysis (Table 2).

### 2.3. Laboratory Analysis

The serum was analyzed for rabies antibody concentration. For this purpose, the rapid fluorescence focus inhibition technique was used, following an adaptation of the method proposed by Zalan et al. [12]. Individual serum samples were evaluated for the greatest dilution of antibody titers that achieved total protection of cells against rabies and are reported as log_2_.

Forages were analyzed according to the AOAC [13]: dry matter, method 930.15; crude protein, method 976.05; ether extract, method 920.39; and mineral matter, method 942.05. The concentration of neutral detergent fiber, acid detergent fiber and lignin were analyzed according to the methodology of Van Soest et al. [14]. The total digestible nutrient content was estimated according to the equations proposed by Weiss et al. [15] and net energy for maintenance and gain according to the equations of NASEM [16].

### 2.4. Statistical Analysis

The experimental design was completely randomized. All analyses were performed using calves as the experimental unit and with the Satterthwaite approximation to determine the degrees of freedom of the denominator of the fixed-effects test. The average daily gain was analyzed by the MIXED procedure of SAS (SAS Inst. Inc., Cary, NC, USA; version 9.4) and included as fixed effects: NF, BAS, and NF × BAS and as random effects: animal (NF × BAS) and sex, and as a covariate: BW on d −14. The other quantitative variables (BW, serum rabies antibody titers concentration, temperament, and pasture behavior variables) were also analyzed using the MIXED procedure of SAS but as repeated measures over time, and they were included as fixed effects: NF, BAS, day and all possible interactions and as random effects: animal (NF × BAS) and sex. The values obtained on d−14 were also included as a covariate for BW and each temperament variable. To determine the most appropriate covariance structure, we compared multiple options, including compound symmetry (CS), first-order autoregressive (AR(1)), unstructured (UN), and Toeplitz (TOEP). The CS structure consistently yielded the lowest Akaike Information Criterion (AIC) values across most repeated-measures models, indicating superior fit in our data. The means were separated using the PDIFF function, and all results were reported as LSMEANS followed by standard error of the mean (SEM). Significance was defined when *p* ≤ 0.05 and tendency when *p* > 0.05 and *p* ≤ 0.10.

## 3. Results

### 3.1. NF Effects

**Figure 1:**
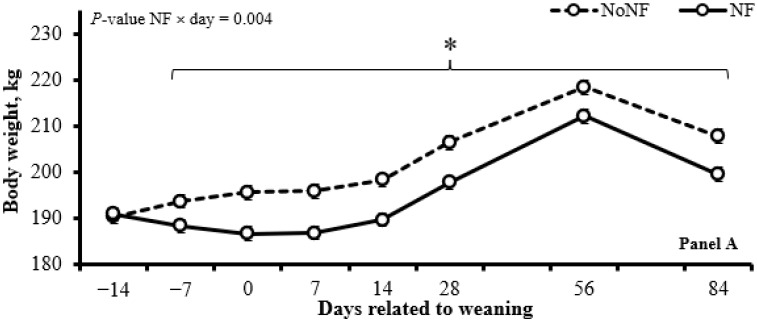

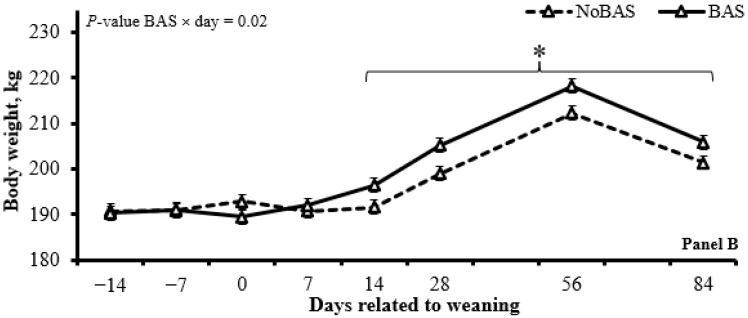
Body weight of calves using a nose flap (NF; Panel A) or administered a bovine-appeasing substance (BAS; Panel B). In a 2 × 2 factorial arrangement, calves were assigned to the 1st factor: use an NF device (Walmur, Porto Alegre, Rio Grande do Sul, Brazil) or not (NoNF) from d −14 to d 0 (weaning) and to the 2nd factor: BAS administration (Secure Cattle, IRSEA Group, Quartier Salignan, France) or not (NoBAS; saline solution; 0.9% NaCl) on d 0. Both BAS and saline solutions were administered topically in the nuchal area in the amount of 5 mL/animal, as recommended by the manufacturer. * *p* ≤ 0.05.

**Figure 2:**
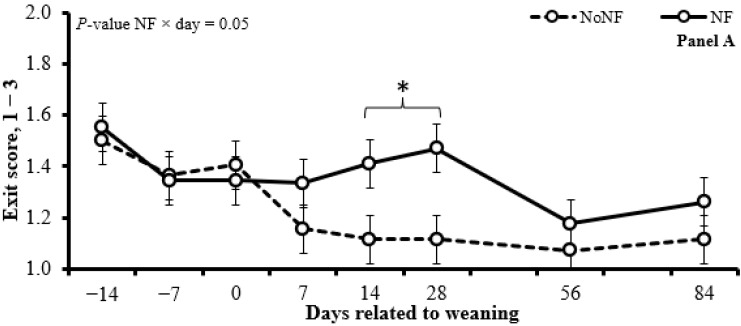

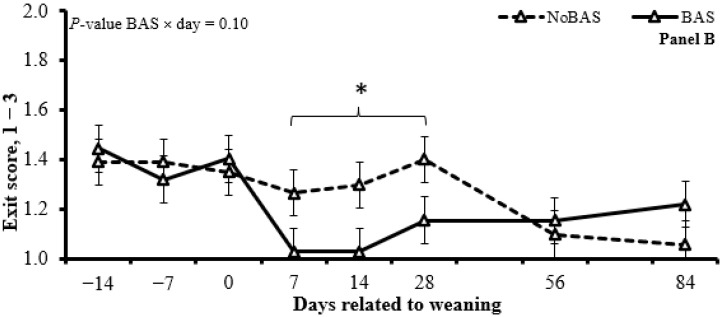
Exit score from the chute of calves using a nose flap (NF; Panel A) or administered a bovine-appeasing substance (BAS; Panel B). In a 2 × 2 factorial arrangement, calves were assigned to the 1st factor: use an NF device (Walmur, Porto Alegre, Rio Grande do Sul, Brazil) or not (NoNF) from d −14 to d 0 (weaning) and to the 2nd factor: BAS administration (Secure Cattle, IRSEA Group, Quartier Salignan, France) or not (NoBAS; saline solution; 0.9% NaCl) on d 0. Both BAS and saline solutions were administered topically in the nuchal area in the amount of 5 mL/animal, as recommended by the manufacturer. * *p* ≤ 0.05.

The use of NFs reduced (*p* ≤ 0.01) the BW and the ADG before (d −14 to 0) and after (d 0 to 84) weaning (Table 3; Figure 1; Panel A). The use of NFs did not affect (*p* ≥ 0.15) the chute score and serum rabies antibody titers concentration (Table 4) but increased (*p* = 0.05) the exit score on d 14 and 28 (Figure 2; Panel A).

**Figure 3:**
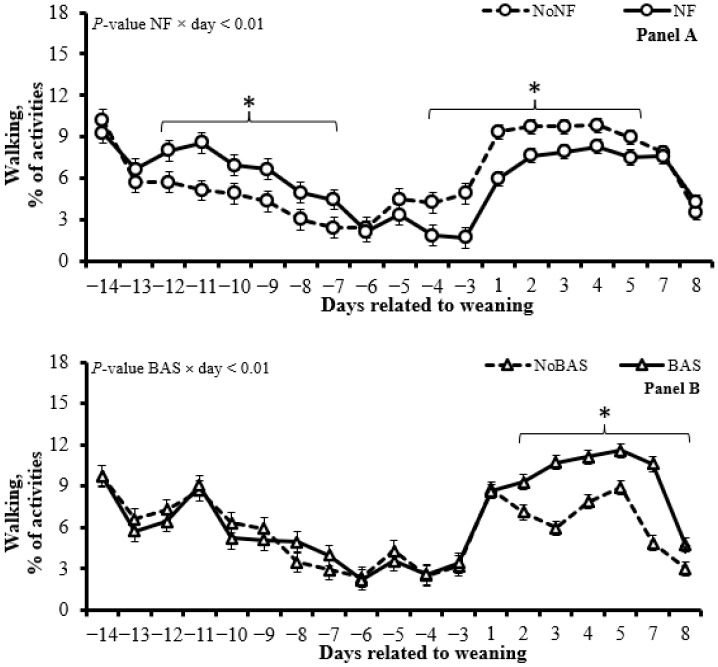

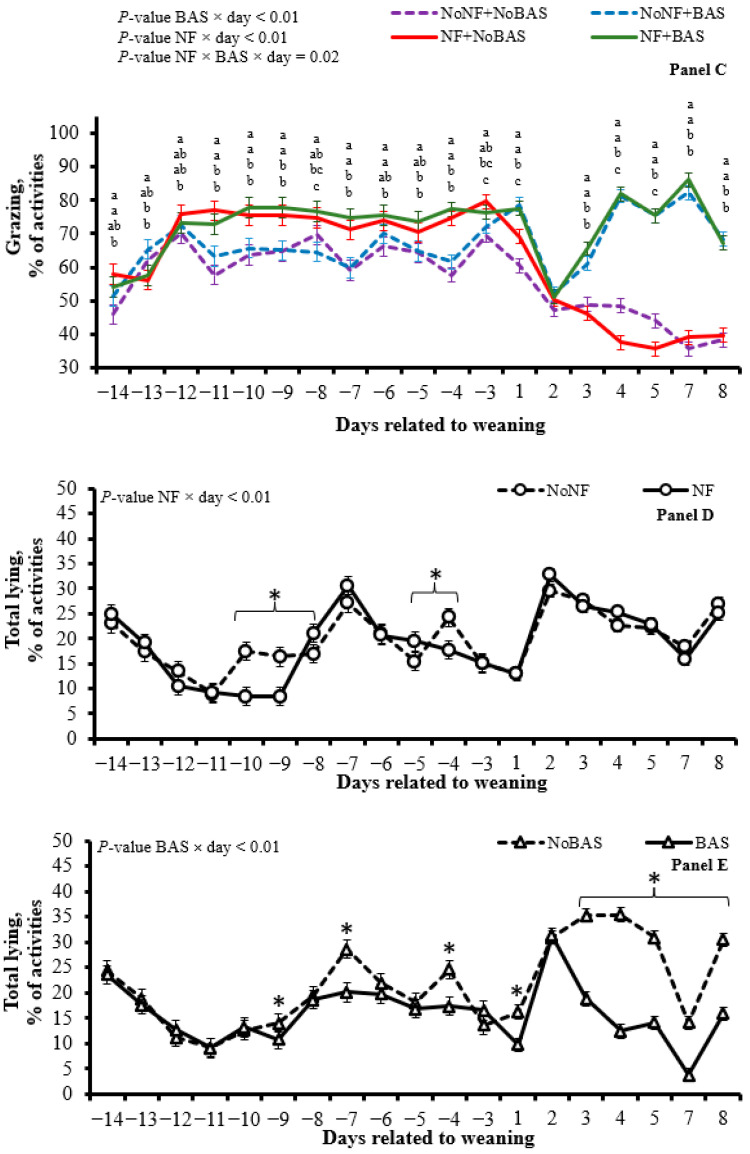

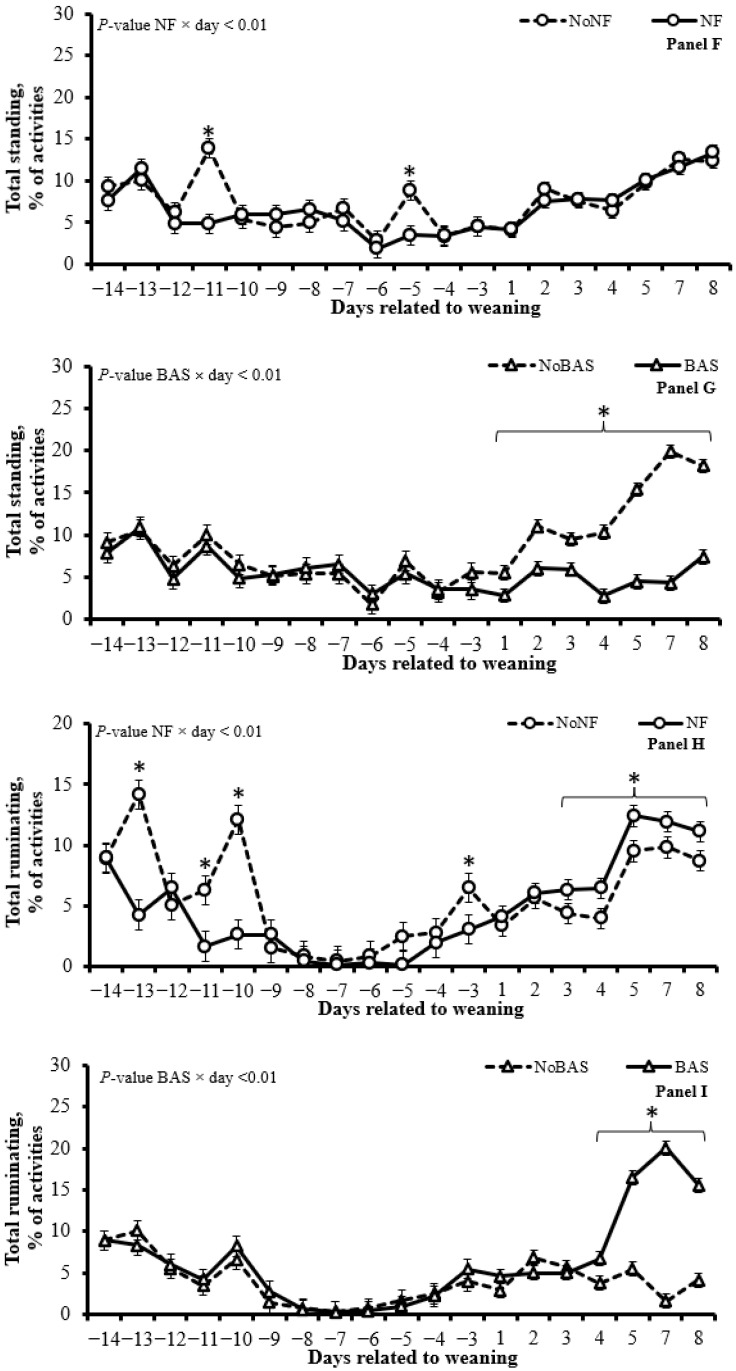

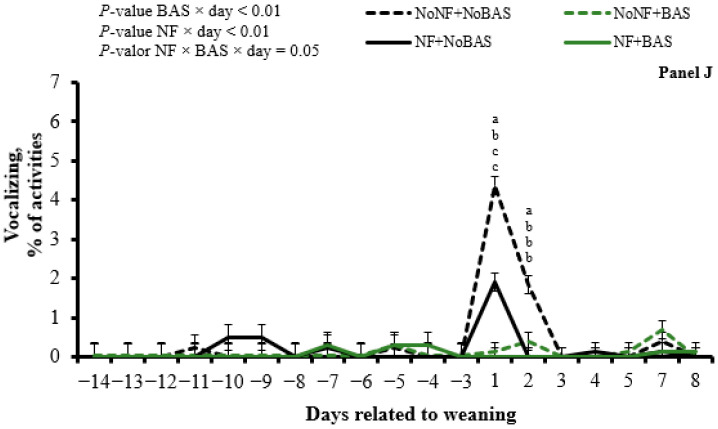
Daily pasture behavior [walking (Panels A and B), grazing (Panel C), total lying (Panels D and E), total standing (Panels F and G), total ruminating (Panels H and I) and vocalizing (Panel J)] of calves using a nose flap (NF) or administered a bovine-appeasing substance (BAS). In a 2 × 2 factorial arrangement, calves were assigned to the 1st factor: use an NF device (Walmur, Porto Alegre, Rio Grande do Sul, Brazil) or not (NoNF) from d −14 to d 0 (weaning) and to the 2nd factor: BAS administration (Secure Cattle, IRSEA Group, Quartier Salignan, France) or not (NoBAS; saline solution; 0.9% NaCl) on d 0. Both BAS and saline solutions were administered topically in the nuchal area in the amount of 5 mL/animal, as recommended by the manufacturer. * or ^a–c^ in the same day represent differences (*p* ≤ 0.05).

The use of an NF increased (*p* < 0.01) the time walking (d −12 to −7; Figure 3, Panel A), grazing (Table 5), total lying (d −8 and −5; Figure 3, Panel D), total standing (d −11 and −5; Figure 3, Panel F) and total ruminating (d 3 to d 8; Figure 3, Panel H) but decreased (*p* < 0.01) the time walking (d −4 to 5; Figure 3, Panel A), lying ruminating (Table 5), total lying (d −10, −9 and −4; Figure 3, Panel D), total ruminating (d −13, −11, −10, and −3; Figure 3, Panel H) and cross-suckling (Table 5).

### 3.2. Effects of BAS

The BAS administration increased (*p* ≤ 0.02) the BW from d 14 to 84 (Figure 1, Panel B) and the ADG from d 0 to 14 (Table 3) and tended (*p* = 0.09) to increase the ADG from d 0 to 84 (Table 3). In addition, the BAS administration tended (*p* = 0.10) to decrease the exit score from d 7 to 28 (Figure 2, Panel B), but it did not affect (*p* ≥ 0.40) the chute score and serum rabies antibody titers concentration (Table 4).

The BAS administration increased (*p* ≤ 0.01) the time walking (d 2 to 8; Figure 3, Panel B), grazing (Table 5), visiting the feed trough (Table 5), and total ruminating (d 4 to 8; Figure 3, Panel I), while it reduced (*p* < 0.01) the time total lying (d −9, −7, −4, 1, 3, 4, 5, 7, 8; Figure 3, Panel E) and total standing (d 1 to 8; Figure 3, Panel G) and tended to decrease (*p* = 0.10) the time vocalizing (Table 5).

### 3.3. NF × BAS Interactions

No interaction (*p =* 0.30) between NF × BAS was detected for BW, but BAS administration tended (*p* = 0.08) to cause a greater increase in ADG from d 0 to 14 in calves that used NF before weaning versus those that did not use (Table 3). No interaction (*p ≥* 0.12) between NF × BAS × day was detected for temperament and response to vaccination (Table 4) nor for most of the pasture behavior variables (Table 5). However, an interaction between NF × BAS × day was detected (*p =* 0.02) for grazing time (after weaning, the use of an NF caused a bigger reduction in grazing time only in calves that did not receive a BAS administration at weaning; Figure 3, Panel C). In addition, an interaction between NF × BAS × day was detected (*p =* 0.05) for vocalizing time (calves NoNF + NoBAS vocalized more than NF + NoBAS that vocalized more than the other treatments on d 1, and calves NoNF + NoBAS vocalized more than all other treatments on d 2; Figure 3, Panel J).

## 4. Discussion

In this study, we hypothesized that the use of NFs from d −14 to d 0, associated with the BAS administration at weaning, would reduce the stress after weaning, improving the growth, immune, and behavior of calves. The use of NFs physically prevents nursing, facilitating a gradual separation from the dam and thereby reducing the abruptness of weaning stress [2]. Consequently, an NF is expected to mitigate stress responses and enhance post-weaning performance in calves. However, the use of NFs reduced the ADG and BW during the entire experimental period (d −14 to d 84). Similar results were described by Burke et al. [17], in which calves using NFs had reduced BW compared to the control group during the seven days preceding weaning. In addition, similar results were observed by Alvez et al. [2], who also observed a reduction in the ADG and BW of calves using NFs. The reduced performance of calves assigned to the NF treatment could be attributed to nasal injuries caused by the NF due to the natural behavior of trying to remove the device. Although nasal injuries were not evaluated in the present study, two previous experiments have reported such occurrences [18,19]. These injuries may limit the intake and increase stress, reducing performance.

Along with the occurrence of local injuries, the use of NFs may predispose calves to face additional health complications. Lippolis et al. [20] described that the weaning method using NFs for 21 consecutive days decreased antibody titer responses after vaccination, suggesting that the devices may trigger a physiological response underlying stress. However, in the present study, the absence of an effect on antibody production following rabies vaccination suggests that the use of NFs did not affect the ability to mount an adequate immune response to vaccination. We acknowledge that assessing only the vaccine-induced antibody response does not provide a comprehensive evaluation of immune function. Future studies should aim to include a broader range of immunological markers, such as acute-phase proteins, cytokines, and leukocyte profiles, to better understand the effects of NFs and BASs on the immune system of calves.

The use of NFs increased the exit scores after weaning (d 14 and 28), and this negative effect on temperament could be attributed to the greater stress caused by wearing the device. Regarding pasture behavior, the greater time spent walking (d −12 to d −7) and grazing (d −11 and d −3) by NF calves can be interpreted as a response to the discomfort caused by the device. However, it is important to highlight that grazing is a natural activity for cattle and can be a coping strategy to deal with stress. In addition, the animals in the NF group exhibited a significant reduction in total lying time (d −10, d −9 and d −4). Such a reduction may indicate a disruption in the normal resting pattern, which may be detrimental to their long-term welfare [10].

The shorter time spent standing in the NF group (d −11 and d −5) suggests a possible association between the use of NF and changes in resting behavior. Furthermore, the decrease in the percentage of total rumination activity during the period before weaning demonstrates that the animals in this group may have experienced some degree of stress or discomfort during this critical transition period [21]. However, on d −2, less vocalization was observed in the NF animals. This behavior represents an important form of communication between cattle, reflecting their emotional state and level of comfort [10]. Thus, the decrease in vocalization observed is a consequence of the reduction in the level of stress or anxiety in the animals in this group, which can be interpreted as a positive adaptation to the use of NFs [22]. Although some previous studies have suggested the use of NFs as an alternative method for the weaning phase, as it provides a reduction in general stress behaviors associated with weaning [23,24], our results demonstrated the opposite behavior.

In contrast to the NF results, BAS administration at weaning tended to increase growth, and it also improved temperament and behavior. The increase in BW observed by BAS administration is consistent with the results obtained by Cooke et al. [7], who evaluated the performance of *B. indicus × B. taurus* beef calves during the weaning phase for 45 d after the administration of BASs and observed a 2.8 kg increase in the BW by BAS administration. Additionally, BASs can exert a favorable influence on the ingestive behavior and digestive efficiency of calves, resulting in a gradual improvement in growth performance over time [3,4,25], which corroborates the observed tendency of an increase in ADG throughout the experimental period.

The observed tendency of lower exit score in the BAS calves between d 7 and d 28 demonstrates that the administration of BASs promotes stress reduction in calves, especially in the first three weeks after weaning, agreeing with previous studies [3,4,26]. The greater walking activity observed between d 2 and d 8 in BAS animals suggests that they were more active after weaning, displaying increased exploratory behavior [3,4], which partially explains the increase in grazing time after weaning.

The significant increase in total rumination activity on d 5 to 8 in BAS calves suggests that BASs may promote a positive response in animal rumination behavior. Rumination indicates gastrointestinal health and welfare in cattle, so an increase in this activity may indicate greater comfort in animals treated with BASs.

The reduction in the percentage of time spent lying by BAS calves suggests a change in the rest and activity patterns of these animals. Although the exact reason for this reduction is not clearly elucidated, it is possible that these animals were more active and engaged in other activities, such as feeding and social interactions [26]. The reduction in time spent can be interpreted as an indication that BAS calves are more relaxed and less likely to be alert or vigilant to potential external stimuli [3]. In addition, the state of relaxation observed in BAS calves is manifested in the decrease in time vocalizing on d 1. Intense vocalization is a typical manifestation in behaviors exhibited by calves after weaning, indicating psychological stress resulting from separation from the dam [10]. Thus, the decrease in vocalization suggests a reduction in the stress response [1,10].

The physiological mechanism through which BAS administration leads to reduced stress is still unclear, but in the literature review, Cappellozza et al. [6] propose the following mechanism: after administration, BASs target organs involved in pheromone perception, including the main olfactory epithelium (MOE) and vomeronasal organ (VNO). The MOE is responsible for the recognition of traditional odor molecules and chemical and environmental signals without specificity or meaning, whereas the VNO is related to pheromone recognition, carrying specific chemosensory signals through the receptors, leading to the occurrence of a neuroendocrine cascade. The VNO neurons can encode stimulus strength, activating an entire neural subpopulation and conducting an electrochemical signal to the calf brain, stimulating the hypothalamus to exhibit an appropriate neuroendocrine response unique to the specific subpopulation of neurons stimulated in the VNO and causing calming effects in the animal.

We recognize that this experiment involved a relatively small sample size (*n* = 24), which may have limited the statistical power to detect subtle differences between treatments.

## 5. Conclusions

Our findings indicate that BAS administration at weaning effectively reduced stress (i.e., demonstrated by lower vocalization and chute exit scores) and enhanced post-weaning growth performance and grazing behavior. In contrast, the use of NFs decreased ADG and increased stress indicators (e.g., exit scores), suggesting it may not be an appropriate strategy for improving weaning outcomes in Nellore calves. Overall, BASs showed clear benefits, whereas NFs alone did not improve, and in some cases worsened, post-weaning responses.

Future studies should evaluate these strategies across different breeds and production systems as well as assess potential long-term effects on animal performance and welfare. Investigating shorter NF use periods, alternative NF designs or brands, and the combined use of BASs with other weaning methods (e.g., fence-line weaning) could provide valuable insights to optimize weaning management practices.

## Figures and Tables

**Table 1:** List of behaviors observed and their respective description.

Items ^1^	Definition
Walking	All four legs were moving with the head raised or not (still)
Drinking water	Mouth below the waterline in the trough ingesting water
Grazing	Picking or consuming pasture with the head close to the ground, still or moving slowly
Visiting the feed trough	Head over the feed trough
Lying down	Lying down in any resting position
Lying down ruminating ^2^	Lying down in any resting position and ruminating
Standing	Not walking
Standing ruminating ^2^	Not walking and ruminating
Cross-suckling	Calf suckling on another
Vocalizing	Making sounds and heard by the observer
Playing	Jumping, running, with no sign of stress

^1^ Adapted from Enríquez et al. [10]. ^2^ Ruminating was defined as chewing regurgitated boluses of feed.

**Table 2:** Chemical composition, forage mass, and forage allowance of pastures (*Urochloa brizantha* cv. Marandu) grazed by calves.

Items ^1^	NF and NoNF Pasture ^2^		NoBAS Pasture ^2^	BAS Pasture ^2^	NF and BAS Pasture ^2^			
	d −14	d 0	d 7	d 7	d 14	d 28	d 56	d 84
CP, g/kg	3.80	4.70	5.70	4.90	6.20	6.30	3.30	4.60
NDF, g/kg	79.6	77.1	69.6	77.8	68.7	66.7	79.8	77.1
ADF, g/kg	69.7	67.4	59.5	69.6	57.3	57.2	71.0	73.8
Lignin, g/kg	5.40	5.40	5.40	6.10	6.10	6.10	6.10	6.10
EE, g/kg	2.00	2.00	1.50	1.20	1.40	1.10	0.80	1.10
Ash, g/kg	8.50	7.90	7.70	7.40	8.40	7.40	8.30	8.80
TDN, g/100 g	56.7	57.0	59.0	55.6	57.1	58.2	53.7	54.2
NEm, Mcal/kg	2.00	2.10	2.10	2.00	2.10	2.10	2.40	2.40
NEg, Mcal/kg	0.60	0.60	0.70	0.60	0.70	0.70	0.50	0.60
FM, kg DM/ha	5040	4640	4392	5368	3840	4560	3720	4800
FA, kg DM/kg of BW	25.9	23.7	22.3	27.2	19.3	22.2	16.9	23.1

^1^ CP, crude protein; NDF, neutral detergent fiber; ADF, acid detergent fiber; EE, ether extract; TDNs, total digestible nutrients; NEm, net energy for maintenance; NEg, net energy for gain; FM, forage mass; DM, dry matter; FA, forage allowance; BW, body weight. ^2^ In a 2 × 2 factorial arrangement, calves were assigned to the 1st factor: use of a NF device (Walmur, Porto Alegre, Rio Grande do Sul, Brazil) or not (NoNF) from d −14 to d 0 (weaning) and to the 2nd factor: BAS administration (Secure Cattle, IRSEA Group, Quartier Salignan, France) or not (NoBAS; saline solution; 0.9% NaCl) on d0. Both BAS and saline solutions were administered topically in the nuchal area in the amount of 5 mL/animal, as recommended by the manufacturer.

**Table 3:** Growth of calves using a nose flap (NF) or administered a bovine-appeasing substance (BAS).

Items ^1^	Treatments ^2^				SEM	*p*-Value ^3^		
	NoNF		NF					
	NoBAS	BAS	NoBAS	BAS		NF	BAS	NF × BAS
BW, kg	199	203	193	194	1.42	<0.01	0.09	0.30
ADG, kg/d								
d−14 to 0	0.378	-	−0.289	-	0.13	<0.01	-	-
d0 to 14	−0.016 ^c^	0.379 ^b^	−0.145 ^d^	0.595 ^a^	0.10	0.66	<0.01	0.08
d14 to 28	0.465	0.698	0.602	0.550	0.13	0.96	0.50	0.29
d28 to 56	0.486	0.567	0.486	0.373	0.57	0.30	0.95	0.18
d56 to 84	−0.404	−0.392	−0.346	−0.522	0.09	0.71	0.39	0.33
d−14 to 84	0.140	0.203	0.089	0.103	0.03	0.007	0.16	0.35
d0 to 84	0.138	0.220	0.070	0.099	0.03	0.003	0.09	0.29

^1^ BW, body weight; ADG, average daily weight gain. ^2^ In a 2 × 2 factorial arrangement, calves were assigned to the 1st factor: use of an NF device (Walmur, Porto Alegre, Rio Grande do Sul, Brazil) or not (NoNF) from d −14 to d 0 (weaning) and to the 2nd factor: BAS administration (Secure Cattle, IRSEA Group, Quartier Salignan, France) or not (NoBAS; saline solution; 0.9% NaCl) on d 0. Both BAS and saline solutions were administered topically in the nuchal area in the amount of 5 mL/animal, as recommended by the manufacturer. ^3^ NF, effect of NF; BAS, effect of BAS; NF × BAS, interaction between NF and BAS. ^a–d^ Different subscripted letters in the same line represent differences (*p* ≤ 0.05) or a tendency to differ (*p* ≤ 0.10).

**Table 4:** Temperament in the chute and serum rabies antibody concentration of calves using a nose flap (NF) or administered a bovine-appeasing substance (BAS).

Items	Treatments ^1^				SEM	*p*-Value ^2^		
	NoNF		NF					
	NoBAS	BAS	NoBAS	BAS		NF	BAS	NF × BAS
Chute score, 1–5	1.63	1.49	1.68	1.68	0.08	0.15	0.40	0.40
Exit score, 1–3	1.16	1.24	1.35	1.23	0.07	0.22	0.77	0.12
Rabies antibody titers ^3^, log_2_	1.76	2.12	3.01	2.23	0.75	0.35	0.78	0.45

^1^ In a 2 × 2 factorial arrangement, calves were assigned to the 1st factor: use an NF device (Walmur, Porto Alegre, Rio Grande do Sul, Brazil) or not (NoNF) from d −14 to d 0 (weaning) and to the 2nd factor: BAS administration (Secure Cattle, IRSEA Group, Quartier Salignan, France) or not (NoBAS; saline solution; 0.9% NaCl) on d 0. Both BAS and saline solutions were administered topically in the nuchal area in the amount of 5 mL/animal, as recommended by the manufacturer. ^2^ NF, effect of NF; BAS, effect of BAS; NF × BAS, interaction between NF and BAS. ^3^ A vaccine against rabies was applied on d −14.

**Table 5:** Daily pasture behavior of calves using a nose flap (NF) or administered a bovine-appeasing substance (BAS).

Items, % of Activities	Treatments ^1^				SEM	*p*-Value ^2^		
	NoNF		NF					
	NoBAS	BAS	NoBAS	BAS		NF	BAS	NF × BAS
Walking	5.68	5.93	5.86	5.61	0.27	0.79	0.99	0.36
Drinking water	1.29	1.21	1.15	1.28	0.14	0.78	0.83	0.45
Grazing	60.6	63.8	65.1	66.6	0.78	<0.01	<0.01	0.30
Visiting the feed trough	0.50	0.72	0.42	0.67	0.08	0.27	0.01	0.88
Lying	16.3	13.5	16.7	14.9	0.67	0.22	<0.01	0.47
Lying ruminating	4.22	5.21	2.90	4.29	0.34	<0.01	<0.01	0.57
Total lying	20.8	18.7	19.7	19.0	0.77	0.66	0.08	0.37
Standing	6.69	5.95	6.04	5.32	0.45	0.16	0.10	0.98
Standing ruminating	0.86	1.06	1.01	1.21	0.13	0.25	0.15	0.98
Total standing	7.60	7.07	7.04	6.64	0.51	0.34	0.37	0.89
Total ruminating	5.07	6.28	3.92	5.51	0.36	0.01	<0.01	0.59
Cross-suckling	2.83	2.38	0.00	0.00	0.15	<0.01	0.15	0.15
Vocalizing	0.31	0.10	0.15	0.06	0.09	0.26	0.10	0.51
Playing	0.08	0.17	0.10	0.08	0.04	0.14	0.73	0.23

^1^ In a 2 × 2 factorial arrangement, calves were assigned to the 1st factor: use an NF device (Walmur, Porto Alegre, Rio Grande do Sul, Brazil) or not (NoNF) from d −14 to d 0 (weaning) and to the 2nd factor: BAS administration (Secure Cattle, IRSEA Group, Quartier Salignan, France) or not (NoBAS; saline solution; 0.9% NaCl) on d 0. Both BAS and saline solutions were administered topically in the nuchal area in the amount of 5 mL/animal, as recommended by the manufacturer. ^2^ NF, effect of NF; BAS, effect of BAS; NF × BAS, interaction between NF and BAS.

## Data Availability

The datasets generated during and/or analyzed during the current study are not publicly available but are available from the corresponding author on reasonable request.
